# *Staphylococcus aureus* small colony variants originating from the lower respiratory tract are associated with persistent pulmonary infection

**DOI:** 10.3389/fimmu.2026.1803861

**Published:** 2026-05-12

**Authors:** Chao An, Shanjian Chen, Anqi Zheng, Lingqi Zheng, Chenshuo Luo, Bin Yang, Yulan Lin

**Affiliations:** 1 Department of Clinical Laboratory, The First Affiliated Hospital, Fujian Medical University, Fuzhou, Fujian, China; 2 Department of Laboratory Medicine, National Regional Medical Center, Binhai Campus of the First Affiliated Hospital, Fujian Medical University, Fuzhou, China; 3 Fujian Key Laboratory of Laboratory Medicine, The First Affiliated Hospital, Fujian Medical University, Fuzhou, China; 4 Clinical Laboratory Diagnostics, The First Clinical College, Fujian Medical University, Fuzhou, China

**Keywords:** biofilm, immune evasion, pulmonary infection, small colony variants, *Staphylococcus aureus*

## Abstract

**Objective:**

*Staphylococcus aureus* small colony variants (SCVs) are responsible for pulmonary infections in cystic fibrosis patients globally, including regions such as Europe and the Americas.There is currently a lack of research in China on persistent pulmonary infections caused by *S. aureus* SCVs. In this study, we aimed to investigate the clinical and pathogenic characteristics of *S. aureus* SCVs in the lower respiratory tract of patients with *Pseudomonas aeruginosa* pneumonia.

**Methods:**

From October 2024 to January 2026, normal phenotype *S. aureus* and *S. aureus* SCVs (characterized by small, slow-growing colonies with atypical morphology) were collected from lower respiratory tract specimens at two tertiary grade A hospitals in Fuzhou. Antibiotic susceptibility testing was performed using the VITEK^®^ 2 Compact system. Molecular characteristics were analyzed by multilocus sequence typing (MLST), virulence genes were screened by PCR, and biofilm formation capacity was assessed using a microtiter plate assay. Pathogenicity was evaluated through serum killing assays and a mouse pulmonary infection model. Patient characteristics were analyzed using the hospital information system.

**Results:**

A total of 46 *S. aureus* SCVs (2.2%) were isolated from 2130 lower respiratory tract specimens from patients with *P. aeruginosa* pneumonia. These SCVs exhibited small colonies, grayish-white color, and reduced hemolysis. More than half of the SCVs were methicillin-resistant *S. aureus* (MRSA), with the predominant genotype being ST1 (19.6%, 9/46). The positive rates of immune evasion-related genes (78.3% vs 53.5%) and the proportion of strong biofilm formation (65.2% vs 39.1%) were significantly higher in SCVs than in normal phenotype *S. aureus*. In addition, SCVs showed greater resistance to host serum and resulted in higher bacterial loads, inflammatory cytokine levels, and pathological damage in the lungs of mice at the late stage (day 28) compared to normal phenotype strains. Notably, patients with SCVs were more frequently treated with fluoroquinolones and mechanical ventilation and had longer hospital stays than those with normal phenotype strains.

**Conclusion:**

*S. aureus* SCVs (often undetected) show a high prevalence of immune evasion genes and a strong ability to form biofilms, and may be associated with persistent pulmonary infection in patients.

## Introduction

*Staphylococcus aureus* is a common clinical pathogen that can cause severe pneumonia, sepsis, septic shock, and other serious complications, potentially endangering patient lives (1). *S. aureus* small colony variants (SCVs) represent a distinct subpopulation of auxotrophic bacteria characterized by small colony size and slow metabolic growth (2). *S. aureus* SCVs are frequently associated with persistent and recurrent infections, including periprosthetic joint infections, infective endocarditis, cystic fibrosis pneumonia, and osteomyelitis (2–5). These SCVs can survive intracellularly within host cells without being eliminated and exhibit enhanced resistance to commonly used clinical antimicrobial agents compared to their wild-type counterparts, rendering clinical management extremely challenging (2).

The formation of *S. aureus* SCVs is primarily attributed to specific auxotrophisms affecting the respiratory chain, including deficiencies in thymidine, menadione, or hemin. These defects impair oxidative phosphorylation, significantly reducing bacterial metabolic rates and ultimately resulting in the SCV phenotype (6). Clinically isolated *S. aureus* SCVs are unstable and prone to reversion to the wild-type phenotype. Consequently, existing research has predominantly relied on stable site-directed mutants (e.g., hemB mutants) to model SCVs. This has limited the study of clinically derived *S. aureus* SCVs, particularly in China.

Current evidence suggests that the formation of *S. aureus* SCVs is closely associated with the presence of *Pseudomonas aeruginosa. P. aeruginosa* facilitates the transition of normal *S. aureus* to respiratory-deficient SCVs through the secretion of 4-hydroxy-2-heptylquinoline-N-oxide (HQNO), a respiratory chain inhibitor that suppresses cytochrome synthesis, impairs electron transport chain function, and slows bacterial growth (7). However, epidemiological data on *S. aureus* SCVs in China remain scarce, especially concerning isolates originating from the lower respiratory tract of patients with *P. aeruginosa* pneumonia. Although numerous studies related to *S. aureus* SCVs have been conducted in China, most consist of isolated case reports. To address this knowledge gap, we hypothesized that *S. aureus* SCVs—which are easily overlooked during routine detection—are present in the lower respiratory tract of patients with *P. aeruginosa* pneumonia. We therefore aimed to examine, via *in vitro* assays, the virulence genes, biofilm formation, antibiotic resistance, and serum resistance of these SCVs, and to evaluate, via *in vivo* mouse models, the inflammatory damage associated with persistent pulmonary infection caused by *S. aureus* SCVs. The findings will provide a theoretical basis for the clinical management of *S. aureus* SCVs infections, enhance clinical understanding of these variants, and hold significant implications for addressing chronic or recurrent pulmonary infections caused by *S. aureus* SCVs.

## Materials and methods

### Bacterial strains

The bacterial strains used in this study were isolated from lower respiratory tract specimens of patients with *P. aeruginosa* pneumonia admitted to two tertiary hospitals in Fuzhou between October 2024 and January 2026. All specimens suspected of harboring *S. aureus* SCVs were streaked onto blood agar plates using the quadrant streaking method to obtain single colonies. Subsequently, the isolates were identified using MALDI-TOF MS (Bruker Daltonik, Germany). Based on colony morphology and growth rate, the strains were classified as either SCVs or wild (normal) phenotype. All normal phenotype *S. aureus* and *S. aureus* SCVs isolated from lower respiratory tract specimens were stored in a -80 °C refrigerator for subsequent experiments.

### Auxotrophism

The auxotrophism of *S. aureus* SCVs was determined as described by Chen et al. (8). Clinical isolates of *S. aureus* SCVs and normal phenotype *S. aureus* controls were inoculated onto Mueller-Hinton agar supplemented with additional thymidine or menadione, as well as onto blood agar plates, to identify thymidine, menadione, and hemin auxotrophy, respectively. The plates were incubated at 35 °C in 5% CO_2_ for 24 hours. Auxotrophy was confirmed by observing whether the SCV colony size reverted to that of the normal phenotype *S. aureus*.

### Clinical data collection

Based on the *P. aeruginosa* pneumonia diagnostic guidelines and the patients’ primary clinical diagnosis information from the hospital information management system, clinical data of hospitalized and outpatient patients with *P. aeruginosa* pneumonia were collected, including patient age, sex, treatment course, and underlying diseases. The data were organized using Microsoft Excel (9).

### Bacterial DNA extraction

DNA was extracted from the collected normal phenotype *S. aureus* and *S. aureus* SCVs using previously described methods (10). Briefly, preserved test strains were streaked onto blood agar plates using the quadrant streaking method and incubated overnight at 35 °C. Subsequently, the bacterial colonies were suspended in 300 μL of sterile distilled water, heated at 100 °C for 10 minutes, and centrifuged at 12000 × g for 5 minutes to remove cellular debris. The supernatant was stored at 4 °C and used as the DNA template for amplification. DNA concentration and purity were measured using a NanoDrop 2000 spectrophotometer. DNA extracted with an A260/A280 ratio of 1.8 to 2.0 and an A260/A230 ratio above 2.0 was considered suitable.

### Multilocus sequence typing

Multilocus sequence typing (MLST) analysis was performed as described previously (11). Seven housekeeping genes of *S. aureus*, namely *arcC* (carbamate kinase), *aroE* (shikimate 5-dehydrogenase), *glpF* (glycerol uptake facilitator protein), *gmk* (guanylate kinase), *pta* (phosphate acetyltransferase), *tpi* (triosephosphate isomerase), and *yqiL* (acetyl-CoA acetyltransferase), were amplified and subjected to Sanger sequencing. The sequencing data were compared with the MLST database (https://pubmlst.org/) to determine the allelic profiles and sequence types (STs) of the *S. aureus* SCVs.

### Virulence and resistance genes

Following methods described in previous studies, polymerase chain reaction (PCR) was used to detect, in the collected normal phenotype *S. aureus* and *S. aureus* SCVs, 15 key virulence genes—including the immune evasion genes *clfA* (clumping factor A), *clfB* (clumping factor B), *fnbA* (fibronectin-binding protein A), *cna* (collagen adhesin), *sdrD* (serine-aspartate repeat protein D), *sdrE* (serine-aspartate repeat protein E), and *ebpS* (elastin-binding protein); the toxin/superantigen genes *sea* (staphylococcal enterotoxin A), *hla* (alpha-hemolysin), *hlg* (gamma-hemolysin), and *pvl* (Panton-Valentine leukocidin); the biofilm formation genes *icaA* (intercellular adhesin gene A), *icaC* (intercellular adhesin gene C), and *icaD* (intercellular adhesin gene D); and the global regulator *sarA* (staphylococcal accessory regulator A)—as well as the resistance gene *mecA* (methicillin resistance gene) (12). The PCR amplification protocol consisted of: initial denaturation at 94 °C for 5 minutes; followed by 30 cycles of denaturation at 95 °C for 30 seconds, annealing at 53 °C for 30 seconds, and extension at 72 °C for 1 minute; and a final extension at 72 °C for 10 minutes. The PCR products were visualized using 2% agarose gel electrophoresis and subsequently sent to Sangon Biotech (Shanghai, China) for sequencing. The sequencing results were analyzed using SnapGene 7.1.2 software.

### Antimicrobial susceptibility testing

Antimicrobial susceptibility testing was performed using the VITEK^®^ 2 Compact system. The results were interpreted according to the Clinical and Laboratory Standards Institute (CLSI) guidelines (13). This study evaluated the susceptibility of the collected normal phenotype *S. aureus* and *S. aureus* SCVs to 13 antibiotics: penicillin, oxacillin, gentamicin, rifampin, ciprofloxacin, levofloxacin, moxifloxacin, clindamycin, erythromycin, linezolid, vancomycin, tetracycline, and tigecycline. *S. aureus* ATCC 25923 was used as the quality control strain. Methicillin-resistant *S. aureus* (MRSA) was confirmed based on the obtained resistance phenotypes and the detection results of the *mecA* gene.

### Biofilm detection

Biofilm formation by the collected 46 strains of normal phenotype *S. aureus* and 46 strains of *S. aureus* SCVs was detected using the microtiter plate method, and the results were averaged over three measurements per replicate per strain (14, 15). Briefly, a single colony of each test strain was inoculated into Tryptic Soy Broth (TSB) and incubated overnight at 37 °C with shaking at 200 rpm. The overnight culture was adjusted to a 0.5 McFarland standard using TSB, and subsequently diluted 1:100 with TSB. Then, 200 μL of the diluted culture was distributed into three wells of a 96-well plate and incubated at 37 °C for 48 hours. Sterile TSB served as the negative control. After incubation, wells were washed three times with phosphate-buffered saline (PBS [pH 7.0]), fixed with methanol solution for 20 minutes, and then the methanol was discarded. The wells were stained with 1% crystal violet solution for 15 minutes, followed by washing with PBS until the wash solution was colorless. After drying, the crystal violet was solubilized with 200 μL of absolute ethanol, transferred to a new microplate, and the absorbance was measured at 570 nm. Mean OD values were calculated for all tested strains and negative controls. The optical density cutoff (ODc) was defined as 3 standard deviations (SDs) above the mean optical density (OD) of the negative control. Classification: non-biofilm producers: OD ≤ ODc; weak-biofilm producers: ODc < OD ≤ 2ODc; moderate-biofilm producers: 2ODc < OD ≤ 4ODc; strong-biofilm producers: OD > 4ODc.

### Serum killing assay

Serum from 10 healthy volunteers was collected, pooled, aliquoted, and stored at -80 °C for subsequent use. Single colonies of the collected normal phenotype *S. aureus* and *S. aureus* SCVs were inoculated into TSB broth and incubated overnight at 37 °C with shaking at 200 rpm. Subsequently, these cultures were subcultured at a 1:200 ratio into fresh TSB broth and incubated for 8 hours at 37 °C with shaking at 200 rpm. The test strains were pelleted by centrifugation at 4 °C, washed with pre-cooled phosphate-buffered saline, and the bacterial concentration was adjusted to 0.5 McFarland standard. Then, 50 μL of the bacterial suspension was mixed with 150 μL of the above-mentioned serum obtained from healthy adult volunteers and incubated at 37 °C with shaking at 60 rpm. Viable bacterial counts were determined at 0 h and 3 h to calculate the survival rate of the test strains at 3 hours.

### Mouse pulmonary infection model

The mouse pulmonary infection model was established based on a previous study with appropriate modifications (16). For the experiments, animals were randomly distributed in a double-blind manner. Briefly, male C57BL/6 mice (6–8 weeks old) purchased from Beijing Huafukang Biotechnology Co., Ltd. were anesthetized with isoflurane. A thin thread was used to hold the mice by their upper incisors, suspending and fixing them on a support. The tongue was carefully extended from the oral cavity using sterile blunt forceps and gently immobilized. The nasal passages were temporarily occluded to maintain oral breathing. Simultaneously, another experimenter used a micropipette to instill 50 μL of a bacterial suspension (0.5 McFarland) prepared from the aforementioned test strains and phosphate-buffered saline (PBS [pH 7.0]) at the junction of the oral cavity and pharynx. Mice in the control group were inoculated with an equal volume of PBS (pH 7.0). Successful pulmonary inoculation was confirmed by a characteristic crackling sound upon reflex inhalation, indicating aerosolization and lung deposition of the inoculum. On days 7, 14, 21, and 28 post-infection, five mice from each group were randomly selected, anesthetized with isoflurane, and euthanized by cervical dislocation. Lung tissues were then collected from the euthanized animals.

### Mouse pulmonary bacterial load

Mouse lung tissues were rinsed in sterile phosphate-buffered saline (PBS [pH 7.0]) under aseptic conditions, and the weight of the lung tissues was recorded using an analytical balance. The left lung of each mouse was separated and placed into a centrifuge tube containing 500 μL of pre-cooled normal saline. The tubes were kept on ice, and the tissues were homogenized using a tissue homogenizer for 5 consecutive cycles, each consisting of 60 seconds of homogenization followed by a 10-second interval. An appropriate amount of the lung tissue homogenate was serially diluted with sterile PBS, and 50 μL of the diluted homogenate was inoculated onto blood agar plates. The inoculum was evenly spread using a sterile cell spreader. After the inoculum had dried, the plates were incubated at 37 °C overnight. Bacterial colonies were enumerated, and the bacterial load in lung tissue was calculated as CFU per gram of tissue. The lung tissue homogenate from each mouse was diluted and subjected to subsequent experiments in triplicate, and the mean colony count was used for result analysis.

### Lung tissue inflammatory cytokines

PCT is a specific indicator for assessing the severity and duration of bacterial pulmonary infection. Its long half-life and high specificity for bacterial infection make it an ideal marker for detecting the inflammatory burden of persistent pulmonary infection caused by SCVs in mice (14, 17). Appropriate amounts of the aforementioned lung tissue homogenates were used to detect mouse interleukin-6 (IL-6), interleukin-10 (IL-10), and procalcitonin (PCT) levels using ELISA kits from Wuhan Elabscience Biotechnology Co., Ltd., according to the reagent instructions. Analysis was based on the average absorbance value obtained from triplicate measurements for each sample.

### Lung tissue pathological sections

The fixed lung tissues were embedded in paraffin after overnight fixation in 4% paraformaldehyde. Sections of 5 μm thickness were prepared and stained with hematoxylin and eosin (H&E) for assessment of pulmonary damage severity (16).

### Statistical analysis

We compared the clinical data of patients with *P. aeruginosa* pneumonia who harbored normal phenotype *S. aureus* and those with *S. aureus* SCVs. Qualitative variables were expressed as percentages, and quantitative variables were expressed as medians with interquartile ranges. Continuous variables were compared using the Mann-Whitney *U* test or *t* ‘test, and categorical variables were compared using Fisher’s exact test or the chi-square test. All statistical analyses were performed using IBM SPSS Statistics 26.0. Statistical significance was set at a *p*-value of <0.05.

## Results

### Prevalence of *Staphylococcus aureus* small colony variants

**Figure 1:**
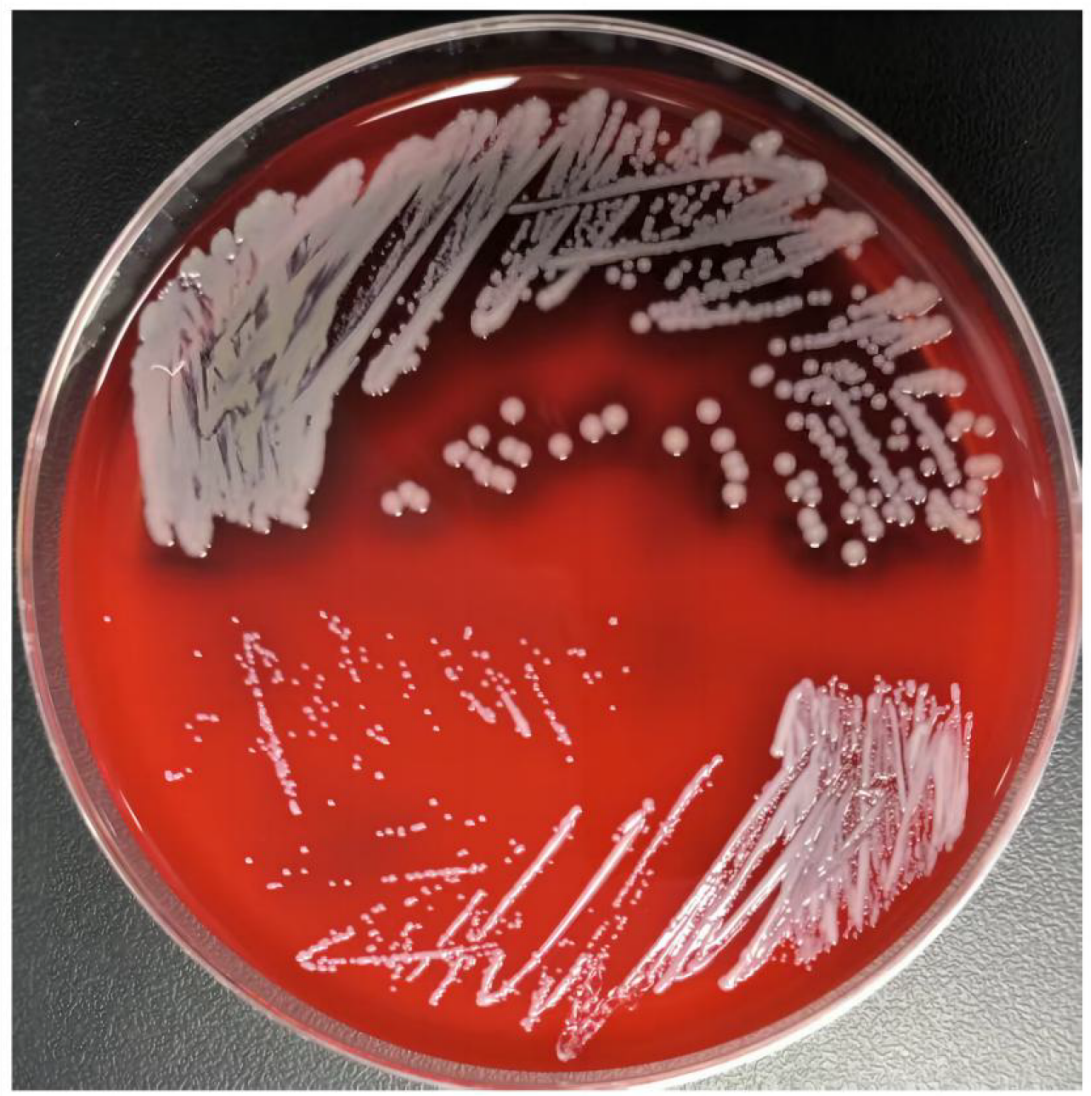
Colony morphology of normal phenotype *S. aureus* and *S. aureus* SCVs derived from the lower respiratory tract. Normal phenotype *S. aureus* (top) and *S. aureus* SCVs (bottom) were cultured on blood agar plates at 37 °C for 24 hours.

A total of 46 *S. aureus* SCVs were isolated from 2130 lower respiratory tract specimens obtained from patients with *P. aeruginosa* pneumonia, yielding a detection rate of 2.2%. As shown in **Figure 1**, when cultured on blood agar plates at 37 °C for 24 hours, *S. aureus* SCVs exhibited marked differences in size, color, and hemolytic capability compared to clinically isolated normal *S. aureus*.

### Auxotrophism

Among the 46 collected isolates, 45 strains were detected as hemin-dependent; no thymidine or menadione auxotrophy was found in any isolate. One additional strain showed no response to any substrate, and its auxotrophism could not be determined. This strain did not exhibit reversion after 15 consecutive passages, and it was subsequently used for follow-up animal experiments.

### Demographic and clinical characteristics

Clinical data of 46 patients with *P. aeruginosa* pneumonia harboring normal phenotype *S. aureus* and those with *S. aureus* SCVs were collected through the Hospital Information System (HIS). Their relevant characteristics were enumerated and compared (**Table 1**). Compared to patients with normal phenotype *S. aureus*, those with SCVs had higher rates of fluoroquinolone use and mechanical ventilation, as well as longer hospital stays (*P* < 0.05). After adjusting for confounding factors, SCVs remained significantly and independently associated with longer hospital stay, longer duration of antibiotic use, increased fluoroquinolone use, and mechanical ventilation (*P* < 0.05).

**Table 1:** Demographic and clinical characteristics.

Variable	Total(n=92)	Patients with *S. aureus* SCVs(n=46)	Patients with normal *S. aureus*(n=46)	P-value*
Demographic features				
Age, years	65(53, 74)	67(53, 76)	63(53, 72)	0.247
Sex, male	54(58.7%)	31(67.4%)	23(50.0%)	0.090
Underlying diseases				
Renal insufficiency	6(6.5%)	2(4.3%)	4(8.7%)	0.677
Hypertension	31(33.7%)	18(39.1%)	13(28.3%)	0.270
Diabetes	17(18.5%)	12(26.1%)	5(10.9%)	0.060
Cancer	6(6.5%)	3(6.5%)	3(6.5%)	1.000
Smoking	12(13.0%)	5(10.9%)	7(15.2%)	0.536
Drinking	8(8.7%)	3(6.5%)	5(10.9%)	0.714
COPD	10(10.9%)	4(8.7%)	6(13.0%)	0.503
CHD	7(7.6%)	5(10.9%)	2(4.3%)	0.434
Skin diseases	4(4.3%)	1(2.2%)	3(6.5%)	0.617
Heart failure	1(1.1%)	0(0.0%)	1(2.2%)	1.000
Thyroid disorder	4(4.3%)	3(6.5%)	1(2.2%)	0.617
Cerebral infarction	9(9.8%)	7(15.2%)	2(4.3%)	0.158
Hyperlipidemia	5(5.4%)	4(8.7%)	1(2.2%)	0.361
Relative examination				
WBC count	7.66(6.29, 9.35)	7.75(6.39,9.01)	7.42(5.92, 10.41)	0.419
LY%	18.0(12.1, 24.2)	19.3(13.2, 26.3)	17.2(9.1, 22.5)	0.121
T > 38°C	9(9.8%)	6(13.0%)	3(6.5%)	0.485
PCT(ng/ml)	0.09(0.05, 0.21)	0.09(0.05, 0.25)	0.09(0.05, 0.19)	0.219
Hb(g/L)	115.5(102.3, 128.0)	117.0(104.5, 130.3)	115.0(95.5, 128.0)	0.648
CRP (nmol/L)	11.3(6.00, 23.43)	7.73(5.10, 14.42)	17.78(8.29, 34.80)	0.062
Blood PMN %	70.8(65.1, 76.4)	70.6(64.8, 75.4)	71.1(66.9, 81.5)	0.085
Clinical treatment and outcomes				
Hospitalization days	19(12, 30)	22(11, 49)	18(13, 28)	0.035
Antimicrobial exposure days	13(6, 22)	17(8,29)	10(5, 18)	0.024
No. (%) using FQ	17(18.5%)	13(28.3%)	4(8.7%)	0.016
Mechanical ventilation%	9(9.8%)	8(17.4%)	1(2.2%)	0.030
Death %	1(1.1%)	0(0.0%)	1(2.2%)	1.000

*Notes: Comparison of patients with *S. aureus* SCVs and patients with normal *S. aureus*.Statistical analysis was performed using Chi square, Fisher’s exact tests or Mann-Whitney *U* tests as appropriate. A *P* value less than 0.05 was statistically significant. Abbreviations: COPD, Chronic Obstructive Pulmonary Disease; CHD, Coronary Heart Disease; WBC, White Blood Cell; LY, Lymphocyte; T, Temperature; PCT, Procalcitonin; Hb, Hemoglobin; CRP, C-Reactive Protein; PMN, Polymorphonuclear Neutrophil; FQ, Fluoroquinolone.

### Multilocus sequence typing

**Figure 2:**
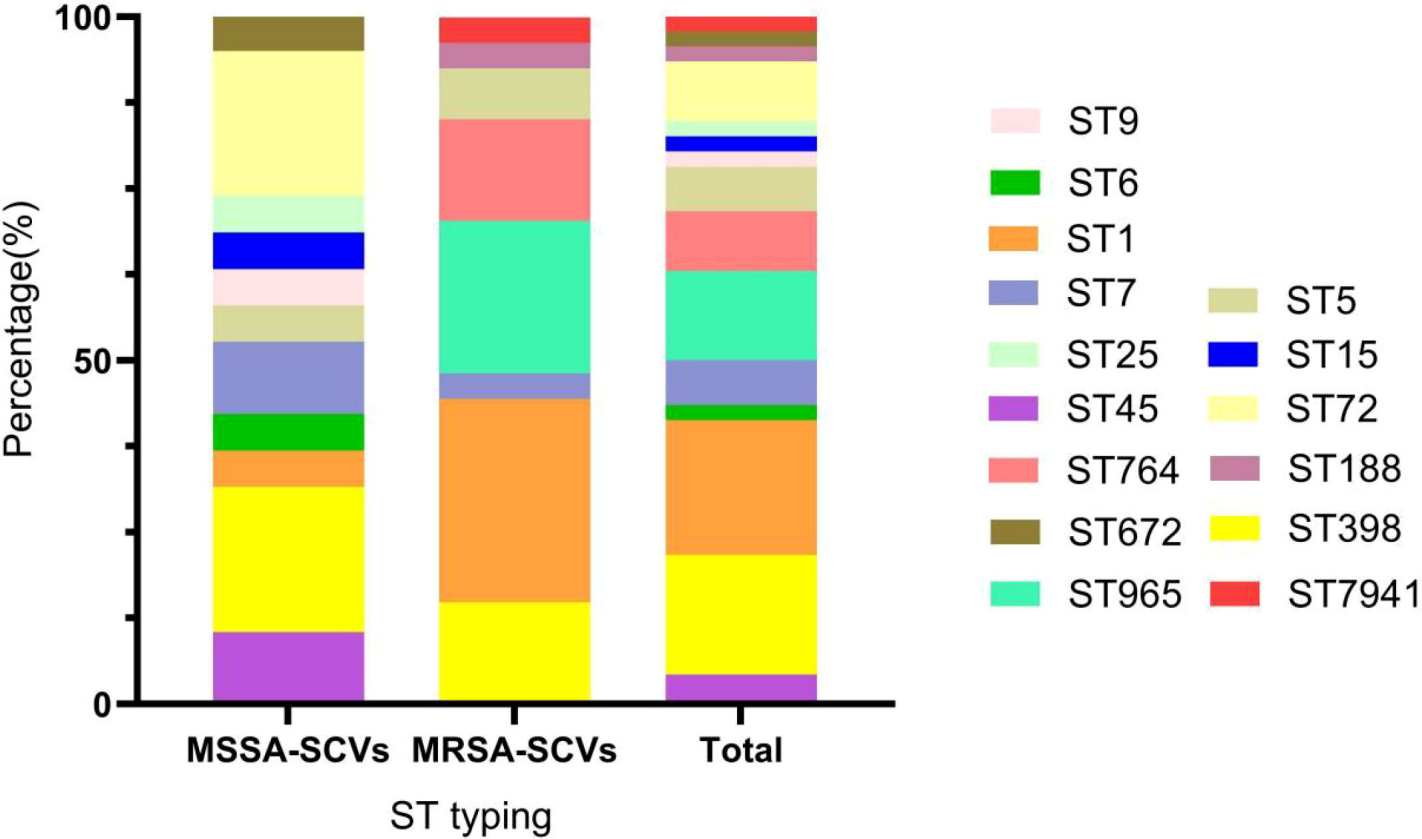
Distribution of sequence types (STs) of all isolates of *S. aureus* SCVs isolates.

Multilocus sequence typing (MLST) analysis of the 46 *S. aureus* SCVs revealed 15 distinct sequence types (STs) (**Figure 2**). Among these, ST1 (19.6%, 9/46) was the predominant clone, followed by ST398 (17.4%, 8/46), ST965 (13.0%, 6/46), ST764 (8.7%, 4/46), and ST72 (8.7%, 4/46). The remaining 10 ST types collectively accounted for 32.6% of the isolates. Among methicillin-resistant *S. aureus* SCVs (MRSA-SCVs), ST1 (29.6%, 8/27) and ST965 (22.2%, 6/27) were the most prevalent, followed by ST398 and ST764 (14.8%, 4/27 each).

### Carriage of virulence genes

The distribution of virulence genes in the isolated normal phenotype *S. aureus* and *S. aureus* SCVs is summarized in **Table 2**. Our results indicate that most *S. aureus* SCVs simultaneously carry multiple virulence determinants, with positivity rates of 100% for the *icaD, hla*, and *hlg* genes, while other virulence genes, including *icaA* and *icaC*, exhibited positivity rates exceeding 80%. Notably, the positivity rates of immune evasion and adhesion/aggregation-related genes *sdrD, sdrE, icaA* and *fnbA* were significantly higher in *S. aureus* SCVs compared to the isolated normal phenotype *S. aureus* (*P* < 0.05).

**Table 2:** Positivity rates of virulence genes in normal phenotype *S. aureus* and *S. aureus* SCVs.

Virulence gene	Total no. (%)	Normal *S. aureus* no. (%)	*S. aureus* SCVs no. (%)	*P-*value*
*sarA*	51(55.4%)	26(57.2%)	25(54.3%)	0.834
*icaA*	73(79.3%)	32(68.6%)	41(89.1%)	0.020
*icaC*	84(91.3%)	39(83.9%)	45(97.8%)	0.059
*icaD*	88(95.7%)	42(91.3%)	46(100.0%)	0.117
*hla*	88(95.7%)	42(91.5%)	46(100.0%)	0.117
*hlg*	87(94.6%)	41(89.5%)	46(100.0%)	0.056
*pvl*	60(65.2%)	32(69.5%)	28(60.9%)	0.381
*sea*	25(27.2%)	9(20.4%)	16(34.8%)	0.101
*fnbA*	82(89.1%)	38(82.6%)	44(95.7%)	0.044
*cna*	61(66.3%)	33(71.1%)	28(60.9%)	0.270
*clfA*	49(53.3%)	28(60.5%)	21(45.7%)	0.144
*clfB*	74(80.4%)	40(86.5%)	34(73.9%)	0.115
*sdrD*	61(66.3%)	25(53.5%)	36(78.3%)	0.015
*sdrE*	46(50.0%)	18(38.9%)	28(60.9%)	0.037
*ebpS*	56(60.9%)	29(63.2%)	27(58.7%)	0.669

*Comparison of the positivity rates of virulence genes in normal phenotype *S. aureus* and *S. aureus* SCVs. Statistical analysis was performed using Chi square or Fisher’s exact tests as appropriate. A *P* value less than 0.05 was statistically significant.

### Antimicrobial susceptibility testing

Results from the VITEK^®^ 2 Compact system revealed that 58.7% of the collected *S. aureus* SCVs were methicillin-resistant *S. aureus*. The isolated *S. aureus* SCVs exhibited high resistance rates to the β-lactam antibiotics penicillin (84.8%) and oxacillin (58.7%). Fortunately, all these strains remained susceptible to linezolid, vancomycin, and tigecycline. Notably, compared to normal phenotype *S. aureus*, SCVs showed significantly higher resistance rates to oxacillin, gentamicin, ciprofloxacin, levofloxacin, moxifloxacin, clindamycin, erythromycin, and tetracycline (*P* < 0.05) (**Table 3**).

**Table 3:** Susceptibility of normal phenotype *S. aureus* and *S. aureus* SCVs to antimicrobials.

Antibiotics	Total			Normal *S. aureus*			*S. aureus* SCVs			P-value*
	S	I	R	S	I	R	S	I	R	
Penicillin	18.5%	0.0%	81.5%	21.7%	0.0%	78.3%	15.2%	0.0%	84.8%	0.420
Oxacillin	52.2%	0.0%	47.8%	63.0%	0.0%	37.0%	41.3%	0.0%	58.7%	0.037
Gentamicin	78.3%	7.6%	14.1%	89.1%	4.3%	6.5%	67.4%	10.9%	21.7%	0.036
Rifampin	97.8%	1.1%	1.1%	100.0%	0.0%	0.0%	95.7%	2.2%	2.2%	1.000
Ciprofloxacin	70.7%	5.4%	23.9%	91.3%	2.2%	6.5%	50.0%	8.7%	41.3%	0.000
Levofloxacin	75.0%	1.1%	23.9%	93.5%	0.0%	6.5%	56.5%	2.2%	41.3%	0.000
Moxifloxacin	76.1%	0.0%	23.9%	93.5%	0.0%	6.5%	58.7%	0.0%	41.3%	0.000
Clindamycin	61.9%	0.0%	38.1%	76.1%	0.0%	23.9%	47.8%	0.0%	52.2%	0.005
Erythromycin	58.7%	1.1%	40.2%	71.7%	2.2%	26.1%	45.7%	0.0%	54.3%	0.006
Linezolid	100.0%	0.0%	0.0%	100.0%	0.0%	0.0%	100.0%	0.0%	0.0%	N
Vancomycin	100.0%	0.0%	0.0%	100.0%	0.0%	0.0%	100.0%	0.0%	0.0%	N
Tetracycline	75.0%	0.0%	25.0%	87.0%	0.0%	13.0%	63.0%	0.0%	37.0%	0.008
Tigecycline	100.0%	0.0%	0.0%	100.0%	0.0%	0.0%	100.0%	0.0%	0.0%	N

*Comparison of antimicrobial resistance rates between normal phenotype *S. aureus* and *S. aureus* SCVs isolates. S, susceptible; I, intermediate; R, resistant; N, no result. Statistical analysis was performed using Chi square or Fisher’s exact tests as appropriate. A *P* value less than 0.05 was statistically significant.

### Biofilm formation capacity

In this study, the biofilm-forming capacity of normal phenotype *S. aureus* and *S. aureus* SCVs was evaluated using the microtiter plate method. Our results demonstrated that the proportion of strong biofilm producers was significantly higher among *S. aureus* SCVs compared to normal phenotype *S. aureus* (*P* = 0.012) (**Table 4**). There was no significant difference in the carriage rate of the intercellular adhesion gene (*ica*) between normal phenotype *S. aureus* and *S. aureus* SCVs within biofilms of the same intensity, suggesting that the high carriage rate of ica may be one of the contributing factors to the strong biofilm-forming capacity of *S. aureus* SCVs.

**Table 4:** Biofilm formation and ica gene carriage in normal *S. aureus* vs *S. aureus* SCVs.

Biofilm and related *ica* genes	Normal *S. aureus* no. (%)	*S. aureus* SCVs no. (%)	*P-*value
None/Weak	9 (19.6%)	3 (6.5%)	0.063
*icaA*	4 (44.4%)	1 (33.3%)	0.636
*icaC*	6 (66.7%)	2 (66.7%)	0.745
*icaD*	7 (77.8%)	3 (100.0%)	0.545
Moderate	10 (41.3%)	13 (28.3%)	0.189
*icaA*	12 (63.2%)	11 (84.6%)	0.178
*icaC*	15 (78.9%)	13 (100.0%)	0.108
*icaD*	17 (89.5%)	13 (100.0%)	0.345
Strong	18 (39.1%)	30 (65.2%)	0.012
*icaA*	16 (88.9%)	29 (96.7%)	0.313
*icaC*	18 (100.0%)	30 (100.0%)	N
*icaD*	18 (100.0%)	30 (100.0%)	N

N, no result. Statistical analysis was performed using Chi square or Fisher’s exact tests as appropriate. A *P* value less than 0.05 was statistically significant.

### Serum killing assay

**Figure 3:**
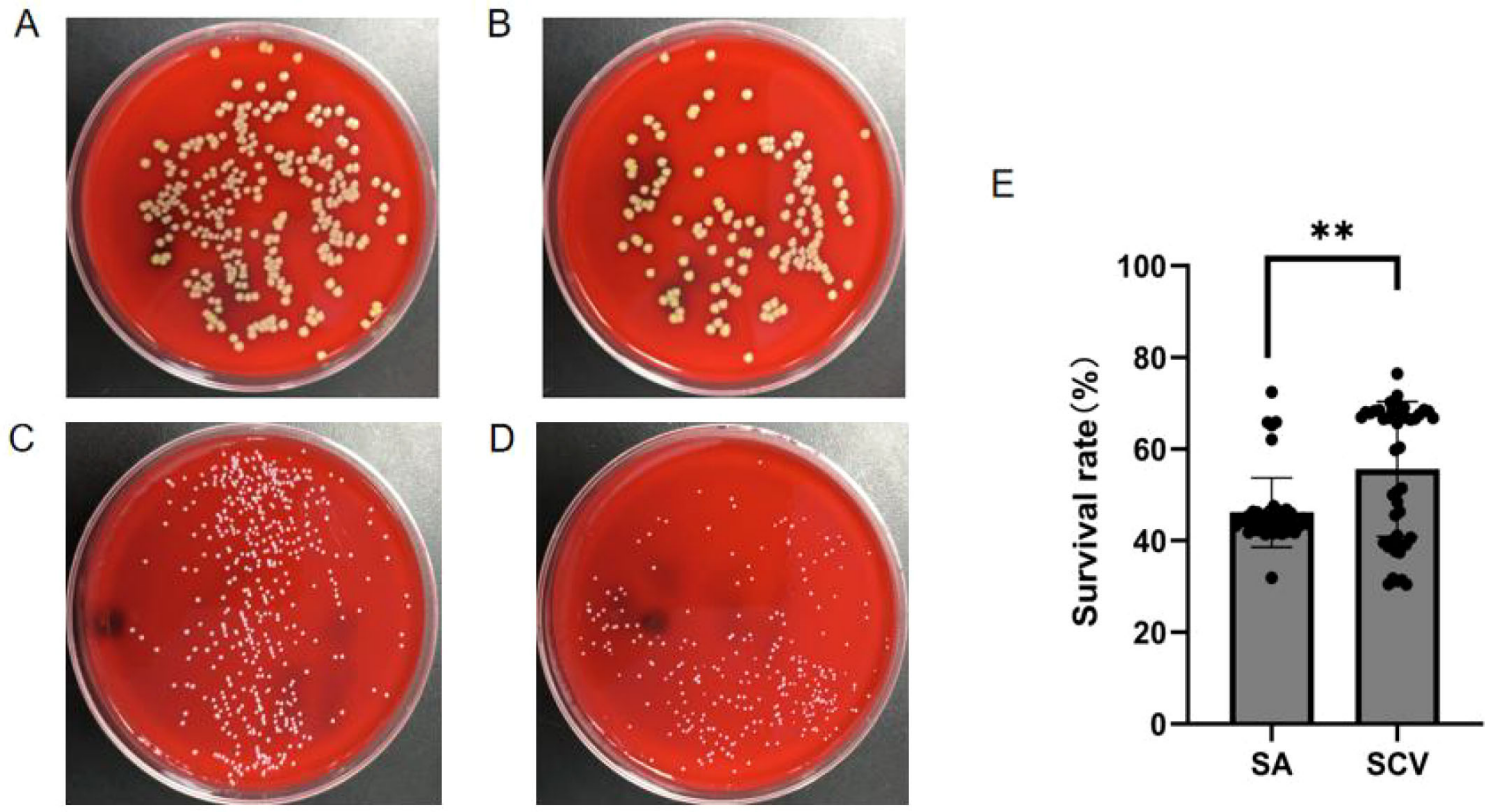
Results of the serum killing assay for normal phenotype *S. aureus* and *S. aureus* SCVs. **(A)** shows colony counts at 0 h for a subset of collected normal phenotype *S. aureus*; **(B)** shows colony counts at 3 h for a subset of collected normal phenotype *S. aureus*; **(C)** shows colony counts at 0 h for a subset of collected *S. aureus* SCVs; **(D)** shows colony counts at 3 h for a subset of collected *S. aureus* SCVs. **(E)** presents the results of the serum killing assay comparing normal phenotype *S. aureus* and *S. aureus* SCVs. N = 46/group (biological replicates). Statistical analyses were performed using Mann-Whitney *U* tests. **P < 0.01.

The serum killing assay evaluates the pathogenicity of microorganisms by assessing their ability to resist complement-mediated killing in serum. In this study, we compared the survival capacity of collected normal phenotype *S. aureus* (SA) and *S. aureus* SCVs (SCV) in healthy human serum using the serum killing assay. Our results demonstrated that SCV exhibited significantly higher serum resistance compared to normal phenotype *S. aureus* (SA) (**Figure 3**).

Additionally, nearly half (3/7, 42.9%) of the *S. aureus* SCVs exhibited significantly higher levels of the immune evasion-related genes (*sdrD, sdrE, fnbA*) tested compared to normal phenotype *S. aureus*. This may be associated with the strong serum resistance of *S. aureus* SCVs (**Table 5**).

**Table 5:** Immune evasion gene carriage rates of normal phenotype *S. aureus* and *S. aureus* SCVs.

Serum resistance and immune evasion genes	Normal *S. aureus* no. (%)	*S. aureus* SCVs no. (%)	*P-*value
Survival rate(%)	44.3 (42.8, 46.3)	66.1 (40.0, 68.1)	0.002
*clfA*	28(60.5%)	21(45.7%)	0.144
*clfB*	40(86.5%)	34(73.9%)	0.115
*cna*	33(71.1%)	28(60.9%)	0.270
*sdrD*	25(53.5%)	36(78.3%)	0.015
*sdrE*	18(38.9%)	28(60.9%)	0.037
*fnbA*	38(82.6%)	44(95.7%)	0.044
*ebpS*	29(63.2%)	27(58.7%)	0.669

Statistical analysis was performed using Chi square or Mann-Whitney *U* tests as appropriate. A *P* value less than 0.05 was statistically significant.

### Mouse pulmonary bacterial load

**Figure 4:**
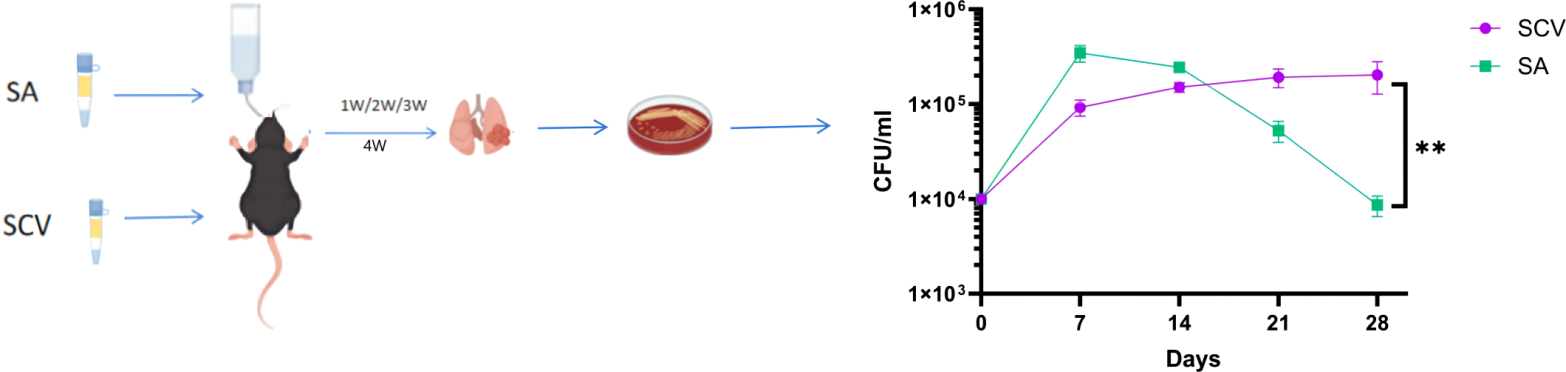
Mouse model of pulmonary infection and results of pulmonary bacterial load. N = 5/timepoint (biological replicates). The difference in CFU was analyzed using *t* ‘test. **P < 0.01.

The mouse pulmonary infection model further simulates the pathological and physiological processes of clinical pulmonary infections. We compared the survival characteristics of normal phenotype *S. aureus* (SA) and *S. aureus* SCVs (SCV) in mouse lungs. Although the pulmonary bacterial load in mice infected with SCV was lower than that in mice infected with normal phenotype SA during the first 14 days, SCV bacterial loads began to exceed those of SA by day 21. At 28 days post-infection, the bacterial load in the SCV group was significantly higher than that in the SA group, demonstrating the survival advantage of SCVs in persistent pulmonary infection in mice (**Figure 4**).

### Mouse pulmonary inflammatory cytokines

**Figure 5:**
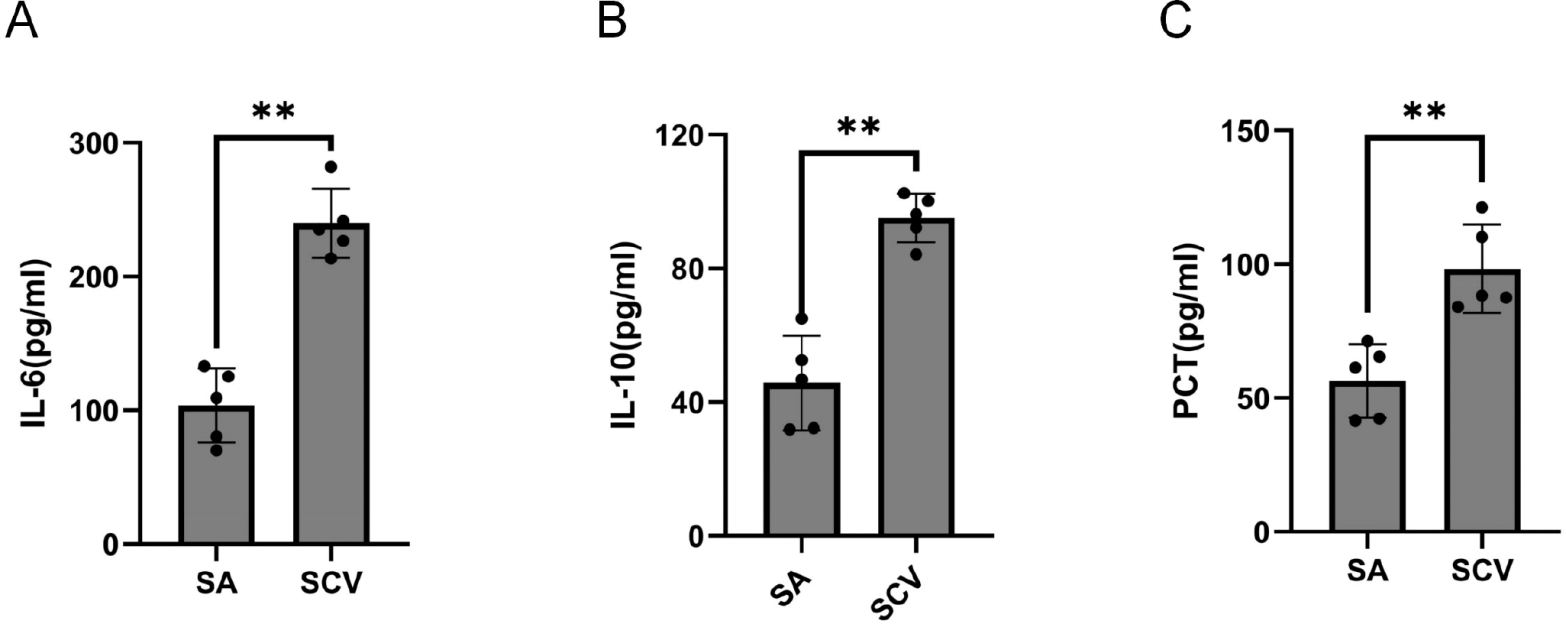
Inflammatory cytokines in lung tissue from mice infected with normal phenotype *S. aureus* (SA) and *S. aureus* small colony variants (SCV). **(A-C)** N = 5/group (biological replicates). *t* ‘test was used for statistical analysis. * *P* < 0.05.

To further investigate the differences in the degree of pulmonary inflammation caused by normal phenotype *S. aureus* (SA) and small colony variants (SCV) during persistent pulmonary infection, we compared the levels of PCT, IL-6, and IL-10 in lung tissues of mice infected with the two groups of strains on day 28 post-infection (**Figure 5**). Our results demonstrated that the pulmonary inflammatory cytokines PCT, IL-6, and IL-10 were significantly higher in mice infected with SCVs compared to those infected with SA. This may be associated with the long-term survival of SCV in the lungs, their high tolerance to antibiotics and immune clearance, and the persistent induction of IL-6, IL-10, and PCT from host immune cells via pathogen-associated molecular patterns (PAMPs) such as cell wall components through pathways like Toll-like receptor 2, thereby creating a dysregulated and protracted inflammatory state.

### Mouse pulmonary pathological damage

**Figure 6:**
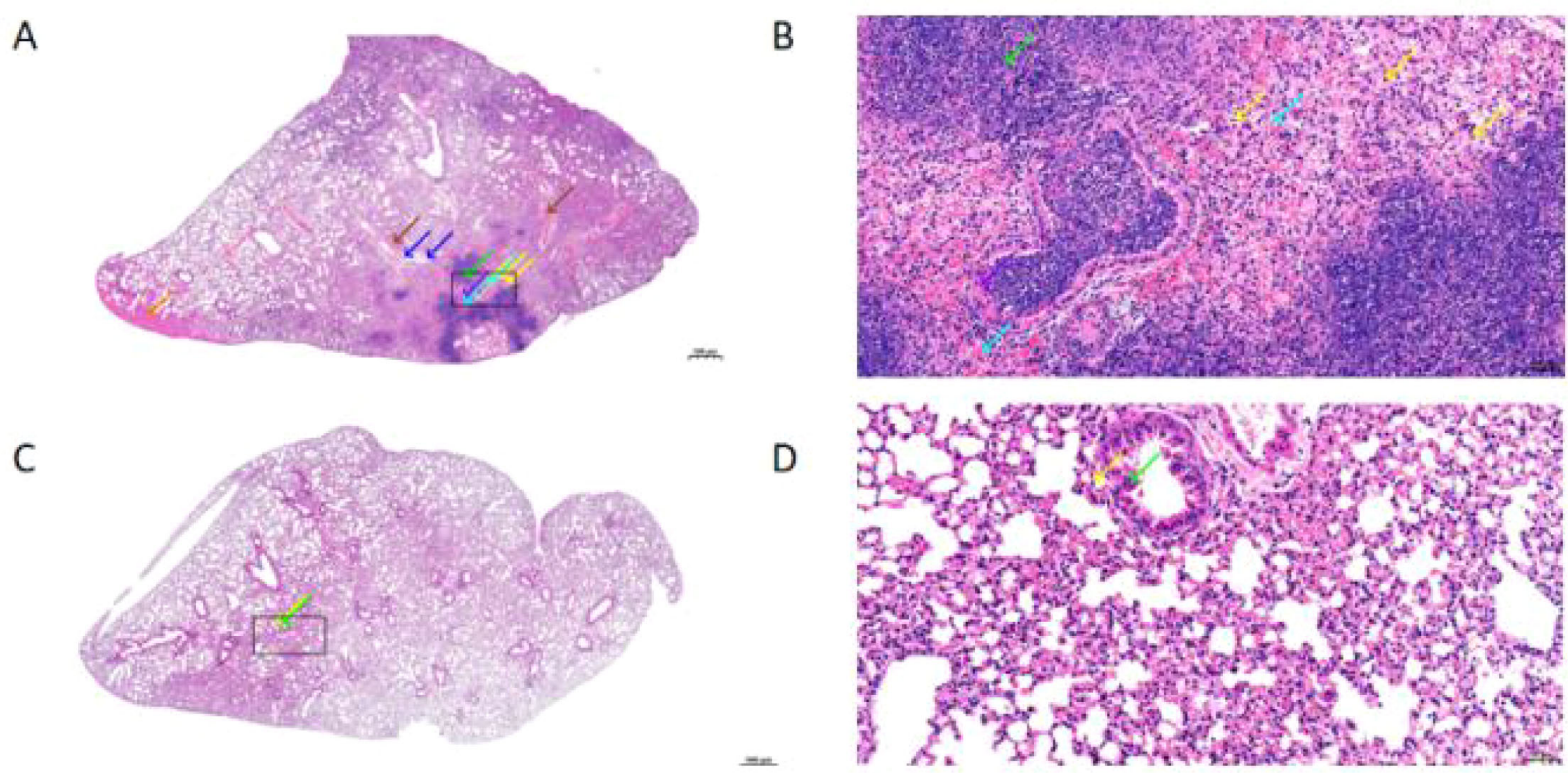
On day 28, the lung tissue (H&E) staining of Staphylococcus aureus small colony variants (SCV) **(A)** and normal phenotype Staphylococcus aureus (SA) **(C)** under low-power field. On day 28, the lung tissue (H&E) staining of Staphylococcus aureus small colony variants (SCV) **(B)** and normal phenotype Staphylococcus aureus (SA) **(D)** under high-power field. Inflammatory cells are indicated by yellow arrows, necrotic cellular debris by green arrows, red blood cells by cyan arrows, eosinophilic material by orange arrows, inflammatory exudate by purple arrows, perivascular edema and hemorrhage by blue and brown arrows respectively, and vascular congestion by pink arrows.

To more comprehensively and intuitively demonstrate the extent of pulmonary inflammatory damage caused by normal phenotype SA and small colony variants (SCV), we performed hematoxylin and eosin (H&E) staining on lung tissues of mice infected with the two groups of strains on day 28 post-infection. Histopathological evaluation of lung tissues from SCV-infected mice revealed moderate areas of pulmonary consolidation, with alveolar narrowing or even disappearance (**Figure 6A**). Extensive infiltration of lymphocytes, granulocytes, and macrophages was observed (yellow arrows); abundant necrotic cellular debris was present (green arrows); widespread alveolar hemorrhage was evident, with red blood cells visible in the alveolar spaces (cyan arrows); focal homogeneous eosinophilic material was observed in the alveolar spaces (orange arrows); mild necrosis of bronchiolar epithelial cells was noted, characterized by pyknotic nuclei and eosinophilic cytoplasm, with necrotic cellular debris, granulocytes, and lymphocyte exudation visible in the lumina (purple arrows); rare vascular necrosis was observed, with eosinophilic material present in the lumina (silver arrows); extensive perivascular edema was evident, with loosely arranged connective tissue and mild infiltration of lymphocytes and granulocytes (blue arrows); perivascular hemorrhage was also observed (brown arrows); and mild interstitial vascular congestion was present (pink arrows) (**Figure 6B**). In lung tissues from mice infected with normal phenotype SA, mild granulocyte infiltration was observed in the alveolar walls (yellow arrows) (**Figure 6D**); there was mild focal thickening of the alveolar walls, widening of the alveolar septa, and compensatory dilation of a few alveoli; mild disorganization of bronchiolar epithelial cells was noted, with shed epithelial cells and eosinophilic material visible in the lumina (green arrows). The black boxes indicate the locations of magnified fields (**Figure 6C**).

## Discussion

*Pseudomonas aeruginosa* pneumonia is one of the most challenging and lethal types of hospital-acquired pneumonia (18). In this study, 46 strains of *Staphylococcus aureus* small colony variants (SCVs) were isolated from 2130 sputum or bronchoalveolar lavage fluid specimens obtained from patients with *P. aeruginosa* pneumonia, yielding a detection rate of 2.2%. Of note, using the same approach, we failed to identify any cases of pure SCVs colonizing the lower respiratory tract in the Chinese population. Globally, the detection rate of *S. aureus* SCVs varies depending on the infection site and geographical region of the study. For example, a study from East Asia identified 19 cases of *staphylococcal* SCVs among 90 patients with positive cultures from cardiac implantable electronic devices (CIEDs), resulting in a detection rate of 21.1% (19); a study from South Asia screened 10 *S. aureus* SCVs from 66 clinical specimens of skin, soft tissue, and bone infections caused by *S. aureus*, corresponding to a detection rate of 15.2% (20); and a study from North America detected 4 *S. aureus* SCVs from pus specimens of 47 patients with diabetic foot ulcers infected with *S. aureus*, with a detection rate of 8.5%. The formation of *S. aureus* SCVs is associated with multiple factors, including iron deficiency or prolonged antibiotic exposure (21, 22). In addition, in our study, auxotroph confirmation experiments showed that 45 out of 46 clinical isolates of *S. aureus* SCVs were hemin-dependent. Clinical SCVs are unstable and prone to revert to the wild phenotype. Currently, in China, clinical isolation and identification of pathogenic bacteria are primarily performed using blood agar plates. This may also explain why clinically derived SCVs are unstable and readily revert to the wild phenotype. Hemin is a key prosthetic group for cytochromes and cytochrome oxidases. When hemin is deficient or its utilization is impaired, the respiratory chain function of *S. aureus* is compromised, leading to a significant reduction in ATP synthesis and slowed growth, thereby resulting in the formation of small colony variants.

*S. aureus* SCVs employ multiple pathogenic mechanisms. Compared to normal phenotype strains, they exhibit enhanced invasive and colonizing capabilities, and can evade host immune responses by inhibiting chemokine expression, residing intracellularly, and escaping immune clearance, thereby rendering them difficult to eliminate (4). In a prospective study of pulmonary infections in 252 cystic fibrosis patients, Silke et al. found that *S. aureus* SCVs demonstrated significantly higher antibiotic resistance rates compared to normal phenotype strains. Furthermore, patients harboring SCVs exhibited more severe disease characteristics, such as reduced forced expiratory volume in 1 second (FEV1) (*P* = 0.0148), compared to those carrying only normal phenotype *S. aureus*. Therefore, in-depth investigation of the clinical characteristics, virulence, resistance genes, and other molecular features of *S. aureus* SCVs is crucial for the prevention and control of such infections (23).

Analysis of clinical data from patients with *P. aeruginosa* pneumonia, collected via the Hospital Information System (HIS), revealed that compared to patients with normal phenotype *S. aureus*, those harboring *S. aureus* SCVs had higher rates of fluoroquinolone use and mechanical ventilation, and significantly prolonged hospital stays and longer duration of antibiotic use (*P* < 0.05). The reason may be related to persistent pulmonary infection and pathological damage caused by *S. aureus* SCVs, although the specific mechanisms require further investigation. Our findings suggest that clinical attention should be paid to pulmonary infections associated with *S. aureus* SCVs.

In this study, the 46 *S. aureus* SCV isolates comprised 15 distinct ST types, indicating considerable genetic diversity. The predominant type was ST1 (19.6%, 9/46), followed by ST398 (17.4%, 8/46). The ST1 lineage of *S. aureus* initially emerged in North America (USA and Canada) and subsequently spread to Europe and Brazil, with no prior reports of its epidemic dissemination in China (24).This may be related to the close trade links between Fuzhou, located in the southeastern coastal region of China, and Brazil and North America in recent years. The ST398 lineage of *S. aureus* is primarily found in animal hosts such as pigs, and it has recently been reported that methicillin-resistant *S. aureus* ST398 can be transmitted to humans through the pork production chain (25). This highlights the importance of paying greater attention to the One Health concept.The pathogenicity of *S. aureus* SCVs is linked to the expression of various adhesins (2). Adhesins promote bacterial adhesion and colonization, facilitating biofilm formation. Biofilms enhance bacterial resistance to antibiotics and aid in evading killing by host immune cells. The intercellular adhesion gene cluster *icaADBC* is involved in biofilm formation, while *icaR* encodes a repressor protein regulating *icaADBC* expression (26). In the present study, the detection rates of *icaA, icaD*, and *icaC* in *S. aureus* SCVs approached 100.0%, whereas in normal phenotype *S. aureus* infections, the rates were 68.6%, 91.3%, and 83.9%, respectively, consistent with findings by Omidi et al. (27). This may explain the enhanced biofilm-forming capacity of *S. aureus* SCVs. Adhesion-related proteins, such as fibronectin-binding protein A (*fnbA*) and clumping factors A and B (*clfA* and *clfB*), are also important factors promoting biofilm formation (28, 29). In the *S. aureus* SCVs isolated in this study, the detection rates of *fnbA, clfA*, and *clfB* were 95.7%, 45.7%, and 73.9%, respectively. Notably, the *fnbA* rate was significantly higher than in normal phenotype *S. aureus* (8). The *cna* gene encodes a collagen-binding protein that mediates bacterial adhesion (30). In this study, no statistically significant difference was observed in the detection rate of *cna* between *S. aureus* SCVs and normal phenotype *S. aureus*. These findings indicate that, compared to normal phenotype *S. aureus, S. aureus* SCVs exhibit higher detection rates of adhesion-related genes. Furthermore, *PVL*, one of the key virulence factors in *S. aureus*, is encoded by the *lukF-PV* and *lukS-PV* genes and can form pores in host cell membranes, leading to cell damage and subsequent leukocyte apoptosis and necrosis (31). The detection rate of *PVL* in the *S. aureus* SCVs isolated in this study was 60.9%, and *PVL* was predominantly found in ST1 *S. aureus* SCVs. This may partially explain the previously reported prevalence of ST1 *S. aureus* (32). The hemolysins encoded by hlgA, hlb, hld, and hla can penetrate host cell membranes, causing target cell lysis (33). In this study, the positivity rates for hlg and hla genes in *S. aureus* SCVs were both 100.0%, higher than the rates of 89.5% and 91.5% observed in normal phenotype *S. aureus*. Notably, the detection rates of the adhesion and immune evasion-related genes *sdrD* and *sdrE* were 78.3% and 60.9%, respectively, significantly higher than in normal phenotype *S. aureus*. The high expression of these adhesins and other virulence factors in *S. aureus* SCVs likely plays a crucial role in the persistent pulmonary infections they cause.

The issue of antibiotic resistance in *S. aureus* cannot be overlooked. The results of this study showed that the detection rate of *mecA* in *S. aureus* SCVs was 58.7% (27/46). The *mecA* gene encodes an alternative penicillin-binding protein, PBP2a. The active site (antibiotic-binding region) of PBP2a has a compact structure, making it difficult for most β-lactam antibiotics, such as penicillins and cephalosporins, to bind stably, thereby conferring resistance to β-lactams and defining methicillin-resistant *S. aureus* (34). In this study, over half of the *S. aureus* SCVs were MRSA, and these strains exhibited significantly higher resistance to quinolone antibiotics, including levofloxacin, ciprofloxacin, and moxifloxacin, compared to normal phenotype *S. aureus*. This may be associated with the unique physiological changes in SCVs, including the active downregulation of their own metabolism, switching from an “aggressive mode” to a “latent mode”. This strategy fundamentally undermines the action basis of antibiotics that depend on the active physiological state of bacteria (such as aminoglycosides and cell wall-active agents), thereby conferring a stronger survival advantage when facing antimicrobial therapy. This is also a major reason why infections caused by SCVs in clinical settings tend to become chronic, recurrent, and difficult to eradicate.It is noteworthy that patients harboring SCVs had higher rates of fluoroquinolone use (**Table 4**), which may contribute to the elevated fluoroquinolone resistance observed in these isolates. Additionally, *S. aureus* SCVs demonstrated a significantly greater capacity for strong biofilm formation, and the proportion of strong biofilm producers was significantly higher than in normal phenotype *S. aureus* (*P* = 0.012), potentially associated with the longer hospitalization stays observed in affected patients.

Finally, we further investigated the infection characteristics of *S. aureus* SCVs using a mouse pulmonary infection model. Compared with normal phenotype *S. aureus*, SCVs persisted in mouse lungs for more than four weeks and exhibited significantly higher bacterial loads during the late stage of infection (**Figure 4**), highlighting their advantage for sustained survival in the host. Moreover, at four weeks post-infection, SCVs showed marked accumulation of the inflammatory cytokines PCT, IL-6, and IL-10 in mouse lungs (**Figure 5**). This elevation of inflammatory mediators indicates that SCVs trigger a persistent and intense inflammatory response. SCVs also caused more severe lung tissue damage at four weeks post-infection, including extensive infiltration of lymphocytes, granulocytes, and macrophages, widespread alveolar hemorrhage, and cell necrosis (**Figure 6B**). The persistently elevated inflammatory response in the SCV group during the late stage of infection suggests a prolonged inflammatory process, and this sustained inflammatory state reflects the ability of SCVs to evade immune clearance. The persistent chronic inflammation induced by SCVs, together with their enhanced tolerance to immune clearance and antibiotics, promotes persistent and refractory infection.

Limitations of the study warrant consideration. Firstly, this study was conducted solely in the Fuzhou region, which may limit the diversity and representativeness of the included bacterial strains. Secondly, we did not perform mechanistic validation such as knockout and complementation of immune evasion-related genes. Additionally, although we established a mouse lung infection model to clarify the pathogenic characteristics of *S. aureus* SCVs, we did not conduct direct animal experiments to validate biofilm and virulence genes. Finally, there is no widely accepted standard to identify SCVs. Based on existing studies, the strains were cultured on blood agar plates for 24 to 72 hours. According to the colony morphology and growth rate, the strains were identified as SCVs or wild phenotype. This may lead to a lack of standardization in our study. Despite these limitations, our findings provide a foundation for a deeper understanding of the clinical and pathogenic characteristics of *S. aureus* SCVs in persistent *P. aeruginosa* pneumonia.

## Conclusion

In summary, this study collected clinical information from patients with persistent *Pseudomonas aeruginosa* pneumonia and performed multilocus sequence typing, antibiotic susceptibility testing, biofilm and virulence gene detection, and mouse pulmonary infection modeling on *Staphylococcus aureus* small colony variants isolated from their lower respiratory tract specimens. We found that these *S. aureus* SCVs were predominantly ST1. Compared to normal phenotype *S. aureus*, *S. aureus* SCVs exhibited enhanced biofilm-forming capacity, a higher carriage rate of immune evasion-related virulence genes, and increased resistance to quinolone and other antibiotics, and were associated with persistent pulmonary infection and damage in mice.

## Data Availability

Data will be available upon reasonable request from the corresponding author (Bin Yang, Email: yangbin2864@163.com).
